# Molecular Epidemiology and Antimicrobial Resistance of Extended-Spectrum *β*-Lactamase (ESBL)-Producing *Klebsiella pneumoniae* in Retail Cattle Meat

**DOI:** 10.1155/2024/3952504

**Published:** 2024-09-21

**Authors:** Nasrin Akter Liza, Hemayet Hossain, Md. Shahidur Rahman Chowdhury, Jarin Al Naser, Rayhan Mahmud Lasker, Asikur Rahman, Md. Ariful Haque, Md. Al Mamun, Md. Mukter Hossain, Md. Mahfujur Rahman

**Affiliations:** ^1^ Department of Medicine Faculty of Veterinary, Animal and Biomedical Sciences Sylhet Agricultural University, Sylhet 3100, Bangladesh; ^2^ Department of Anatomy and Histology Faculty of Veterinary, Animal and Biomedical Sciences Sylhet Agricultural University, Sylhet 3100, Bangladesh

## Abstract

Enterobacteriaceae that produce extended-spectrum *β*-lactamases (ESBLs) can result in severe human infections, contributing to the development of complex diseases. *Klebsiella pneumoniae* is one of the ESBL-producing pathogens that helps to set antimicrobial resistance as a major public health problem worldwide. The current study aimed to isolate, identify, and characterize ESBL-producing *K. pneumoniae* and their antimicrobial resistance pattern in retail cattle meat samples. A comprehensive set of 225 cattle meat samples was gathered from 13 upazilas within the Sylhet district of Bangladesh. The bacterial isolates were obtained through biochemical and cultural techniques, and the identification of *K. pneumoniae* was accomplished using polymerase chain reactions (PCRs). Antimicrobial susceptibilities were assessed using disk diffusion in accordance with the Clinical and Laboratory Standards Institute (CLSI, 2020) guidelines. Genes encoding ESBL enzymes were detected by the double-disk synergy test (DDST) and multiplex PCR. The overall prevalence of *Klebsiella* spp. was 28.89% (65/225), whereas the positive percentage of *K. pneumoniae* was 59.2% (29/49) confirmed by PCR. Antimicrobial resistance was observed against 12 antibiotics. According to the phenotypic resistance pattern determined through the disk diffusion method, all isolates (100%) were resistant to ampicillin, amoxicillin, cefuroxime, cefotaxime, and colistin. On the other hand, the highest susceptibility was observed towards gentamicin (97.95%), followed by ciprofloxacin (85.71%), tetracycline (83.67%), and trimethoprim-sulfamethoxazole (81.63%). Out of the total *K. pneumoniae* isolates analyzed, ESBL genes were present, and the highest percentage, 82.8% (24/29), tested positive for *bla*_TEM_ genes. Interestingly, among the nine ESBL genes, six were identified in *K. pneumoniae* isolates, except for *bla*_OXA,_ *bla*_CTX‐M‐grp2_, and *MultiCase*_DHA_. The study's results reveal the presence of extended-spectrum *β*-lactamase (ESBL)-producing multidrug-resistant (MDR) *K. pneumoniae* in retail cattle meat samples posing a substantial public health threat.

## 1. Introduction

Livestock holds an indispensable role in ensuring food security, balanced nutrition, and alleviation of unemployment through fostering self-employment, generating foreign exchange, enhancing agricultural land fertility, and empowering women in Bangladesh [1]. The livestock sector's contribution to Bangladesh's GDP in FY 2021-22 has experienced an average annual growth rate of 5.39 percent over the past five years [2]. Presently, an individual in Bangladesh consumes 120 grams of meat daily, which is provided by the nation's livestock industry. As per the Bangladesh Ministry of Livestock, the country is currently self-sufficient in livestock, particularly meat production. This signifies that meat production within the nation has persistently increased each year over the past decade. In FY 2021-22, approximately 9.3 million (92.65 lakh) metric tons of meat were produced in Bangladesh [2]. Despite its expansion, the cattle meat industry faces significant challenges due to the irrational application of antibiotics in treatment and prophylaxis. The global food animal sector accounts for over 50% of antibiotic consumption, with a projected 50% increase in antibiotic usage in agriculture by 2030. Annually, approximately 25 million pounds of antimicrobials are utilized for nontherapeutic purposes in livestock, particularly in cattle [3, 4]. The indiscriminate use of antibiotics in cattle farming for treatment and disease prevention exerts substantial selection pressure on bacterial pathogens, potentially fostering the emergence and propagation of antibiotic-resistant bacteria, which the World Health Organization identifies as one of the foremost global public health threats of the 21st century [5, 6]. The primary culprits behind the rise in antibiotic-resistant bacterial infections are organisms producing extended-spectrum *β*-lactamase (ESBL) [7].

The extended-spectrum *β*-lactamase (ESBL) enzyme adeptly degrades penicillin, cephalosporins, and aztreonam, but its function can be obstructed by *β*-lactamase inhibitors such as clavulanic acid [8]. ESBLs have emerged as a major factor in antibiotic resistance within *Enterobacteriaceae*, particularly in *Klebsiella* species. Beta-lactam antibiotics are extensively utilized in veterinary medicine due to their high specificity, ideal selective toxicity, and potent bactericidal effects [7]. Consequently, the overuse of these antibiotics in veterinary practices has exacerbated the emergence and spread of genetic resistance determinants, particularly in *K. pneumoniae* species. The presence of ESBL-producing bacteria in food sources is particularly concerning because these enzymes can be easily transferred between bacteria through plasmids, facilitating the spread of resistance [9]. Plasmidic AmpC *β*-lactamases provide resistance to cephamycins and are not inhibited by *β*-lactamase inhibitors, complicating treatment options further. Carbapenemases, another group of *β*-lactamases, degrade carbapenems, which are often considered the last line of defense against multidrug-resistant bacterial infections [10]. The spread of these resistance mechanisms significantly limits therapeutic options and increases the risk of treatment failures.

*K. pneumoniae* is frequently identified as harboring ESBL-encoding genes, including *bla*_CTX‐M_,  *bla*_SHV_,  and *bla*_TEM_ [11]. In addition, extended-spectrum *β*-lactamase (ESBL)-producing *K. pneumoniae* can contaminate cattle meat when operating procedures and hygiene practices in slaughterhouses and farms are not followed precisely [12]. The prevalent rise of multidrug-resistant (MDR) *K. pneumoniae* poses a significant public health challenge [13]. Infections caused by MDR *K. pneumoniae* are associated with increased mortality rates, prolonged hospitalizations, and elevated healthcare expenditures [14, 15]. Furthermore, recent data indicate an escalating trend of antibiotic resistance in instances of community-onset infections [13, 16, 17]. Within the healthcare setting, *K. pneumoniae* infections are particularly problematic for newborns and old people. For eradicating the threat posed by *K. pneumoniae* to humans, it is necessary to have a solid understanding of the probable source of infecting organisms and to conduct extensive surveillance. Products derived from the animals have the potential to expose people to germs, resistant to antibiotics [18, 19].

The pervasive administration of antibiotics to enhance animal productivity contributes to a milieu wherein bacteria harboring resistance genes can disseminate into the environment via raw meat, posing substantial epidemiological ramifications for human health. This study delineates the antimicrobial resistance profiles of ESBL-producing *K. pneumoniae* isolated from cattle meat procured randomly from retail markets in the Sylhet district of Bangladesh.

## 2. Materials and Methods

### 2.1. Study Design and Sampling Strategy

**Figure 1:**
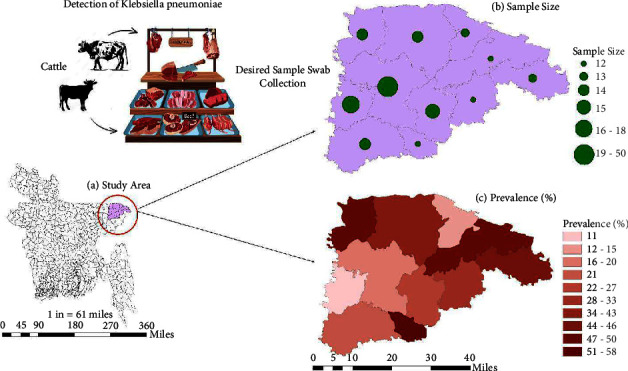
Study area map showing the location of the selected study area, sample size, and prevalence of *K. pneumoniae* isolated from cattle meat in the Sylhet district of Bangladesh.

Between June 2022 and August 2022, a cross-sectional investigation was conducted in 13 subdistricts (upazilas) located within the Sylhet district of Bangladesh. The selected upazilas included Balaganj, Beanibazar, Biswanath, Companiganj, Dakshin Surma, Fenchuganj, Golapganj, Gowainghat, Jaintapur, Kanaighat, Osmani Nagar, Sylhet Sadar, and Zakiganj. The geographic coordinates for these upazilas ranged from 24°36′ to 25°11′ north latitude and 91°38′ to 92°30′ east longitude, as depicted in Figure 1. The samples were collected following a convenient sampling method based on the availability of retail cattle meat shops. To estimate prevalence, the required minimum sample size was calculated using an equation from Mahen et al. [20] and Asha et al. [21].

(1) $$\begin{array}{c} n=\frac{Z^{2}P_{\exp}\left(1-P_{\exp}\right)}{d^{2}}, \end{array}$$

where *P*_exp_ = expected prevalence, *d* = desired absolute precision, and *z* = 1.96 for 95% confidence interval level. *P*_exp_ = 0.16 was used to maximize the sample size based on the previous meta-analysis in Nepal where the prevalence of *K. pneumoniae* was determined as 16% [22]. Using this *P*_exp_ with a desired absolute precision of *d* *=* *0.05,* a required sample size of at least 207 was determined. This study investigated a total of 225 cattle meat samples to detect ESBL-producing *K. pneumoniae*.

### 2.2. Sample Collection and Bacterial Isolation

A total of 225 swab samples were collected from diverse retail shops in the Sylhet district, originating from cattle meat. The swab samples from the cattle meat were collected aseptically and placed in sterile plastic containers with buffered peptone water (BPW; HiMedia Laboratories Pvt. Ltd., Mumbai, India) in the ratio of 1 : 10 and incubated the medium at 37°C for 24 ± 2 hours. Preenriched culture from BPW, a loop of enriched broth, was initially streaked on EMB agar and incubated at 37°C for 24 ± 2 hrs. Circular, dome-shaped, mucoid, pink to purple colored translucent or opaque colonies were found. Following the enrichment, a loop of enriched broth from EMB media was streaked on MacConkey agar and incubated at 37°C for 24 ± 2 hrs for selective enrichment of *Klebsiella* spp. colonies (single mucoid, pink to red-colored opaque). Positive colonies from the MacConkey plate were further inoculated in a nutrient agar plate for pure colonies and incubated for 24 ± 3 hrs at 37°C. Presumptive identification of *Klebsiella* spp. was performed by biochemical tests. The biochemical tests included the sugar fermentation test; citrate test; methyl red Voges–Proskauer test; and motility, indole, and urease test.

### 2.3. Identification of *Klebsiella* spp

The genomic DNA of *Klebsiella* isolates was extracted according to the manufacturer's instructions using a DNA extraction kit (AddBio Inc. Ltd., Daejeon, Korea). Two sets of reference primers were chosen to conduct multiplex PCR to amplify the target genes of the genus *Klebsiella* and *K. pneumoniae*. For all reactions, the amplification conditions comprised an initial denaturation step at 95°C for 5 minutes in one cycle. This was succeeded by 35 cycles involving denaturation at 95°C for 60 seconds, annealing at 55°C for 60 seconds, and extension at 72°C for 2 minutes. The final extension was carried out at 72°C for 10 minutes [23]. The primer sequences are detailed in Table 1.

### 2.4. Antimicrobial Susceptibility Testing

The antimicrobial susceptibility of *Klebsiella* isolates was assessed through the Kirby–Bauer disk diffusion method on Mueller–Hinton agar [26] and then the diameter of the inhibition zone was measured in accordance with the Clinical and Laboratory Standards Institute (CLSI) guidelines from 2020 [27]. The assay was performed with 12 different antimicrobial agents, including ampicillin (AMP, 10 *μ*g), amoxicillin (AMX, 10 *μ*g), gentamicin (GEN, 10 *μ*g), amikacin (AK 10 *μ*g), cefuroxime (CXM, 30 *μ*g), ceftriaxone (CTR, 30 *μ*g), cefotaxime (CTX, 30 *μ*g), tetracycline (TE, 30 *μ*g), ciprofloxacin (CIP, 5 *μ*g), colistin (CL, 30 *μ*g), trimethoprim-sulfamethoxazole (COT, 1.25/23.75 *μ*g), and aztreonam (AZ, 10 *μ*g).

### 2.5. Double-Disk Synergy Test (DDST)

The double-disk synergy test (DDST) method was utilized to detect ESBL production in *K. pneumoniae* isolates. The procedure was performed as follows: first, a bacterial suspension equivalent to a 0.5 McFarland standard from each isolate was prepared as described by Hoque et al. [28]. This suspension was then uniformly spread onto the surface of Mueller–Hinton agar plates using a sterile cotton swab. An amoxicillin-clavulanic acid (30 *μ*g) disk was placed in the center of the agar plate. Subsequently, ceftriaxone (30 *μ*g) and cefotaxime (30 *μ*g) disks were positioned 20 mm (center to center) from the amoxicillin-clavulanic acid disk. The plates were incubated at 37°C for 24 hours. After incubation, the plates were examined for the characteristic “keyhole” or synergy effect, which indicates ESBL production. This effect is observed as an enhanced zone of inhibition between the cephalosporin disks and the amoxicillin-clavulanate disk, suggesting that the *β*-lactamase inhibitor (clavulanate) restored the activity of the cephalosporins.

### 2.6. Molecular Detection of ESBL Genes

PCR screening was conducted on all *Klebsiella* isolates to detect ß-lactamase genes, including *bla*_TEM_, *bla*_SHV_, *bla*_OXA_ *bla*_CTX‐M‐grp1_, *bla*_CTX‐M‐grp2_, *bla*_CTX‐M‐grp9_, *MultiCase*_ACC_, *MultiCase*_MOX_,  and MultiCase_DHA_ 12.5 *μ*L of 2x master mix (AddBio Inc, South Korea), 0.5 *μ*L each of forward and reverse primers (10 pmol/*μ*L), and 9.5 *μ*L of nuclease-free water. The multiplex PCR amplification for ß-lactam gene detection involved an initial denaturation at 95°C for 5 minutes, followed by 30 cycles of denaturation at 94°C for 30 seconds, annealing at 62°C for 90 seconds, and extension at 72°C for 1 minute. The amplification concluded with a final elongation step at 72°C for 10 minutes [25].

### 2.7. Statistical Analysis

The information was assimilated, categorized, and structured into Excel spreadsheets. The prevalence was calculated using the formula described by Emon et al. [29]:

(2) $$\begin{array}{c} \text{prevalence}=\frac{\text{number of current cases}\left(\text{new and preexisting}\right)\text{at }a\text{ specified point in time}}{\text{population at the same specified point in time}}\times 100. \end{array}$$

We conducted the chi-square test to assess the associations among various explanatory variables. In cases where the expected count in a cell was less than 5 and occurred in at least 20% of the cells, Fisher's exact test was applied. Pearson's correlation coefficient was performed to estimate the association among the positive ESBL genes and ESBL producer isolates using R (RStudio 4.3.3). Confidence intervals were calculated using the binomial exact test, and a significance level of less than 0.05 was chosen for determining statistical significance. The data analysis was carried out using SPSS version 26.

### 2.8. Geospatial Mapping and Plot

The study area mapping was generated using ArcMap 10.8 (ArcMap 10.8, Esri, USA), utilizing a shapefile extracted from the following website: https://www.diva-gis.org. These data were employed to create both choropleth and dot maps, effectively visualizing the prevalence of some explanatory variables as well as the corresponding sample sizes. In addition, to illustrate the antimicrobial properties of isolates, we employed OriginPro (https://www.originlab.com) and utilized the heat map and Venn diagram file exchange format. This allowed the creation of informative heat maps and Venn diagrams offering a comprehensive view of the data. We also used the Sankey diagram for representing the whole scenario of the research work.

## 3. Results

**Figure 2:**
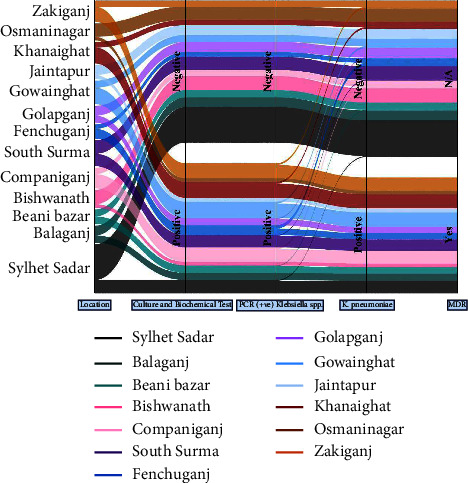
Sankey diagram showing the whole overview of study data, outcomes, and relationship between explanatory variables and outcome variables (*K. pneumoniae* positive and negative and MDR yes/no).

**Figure 3:**
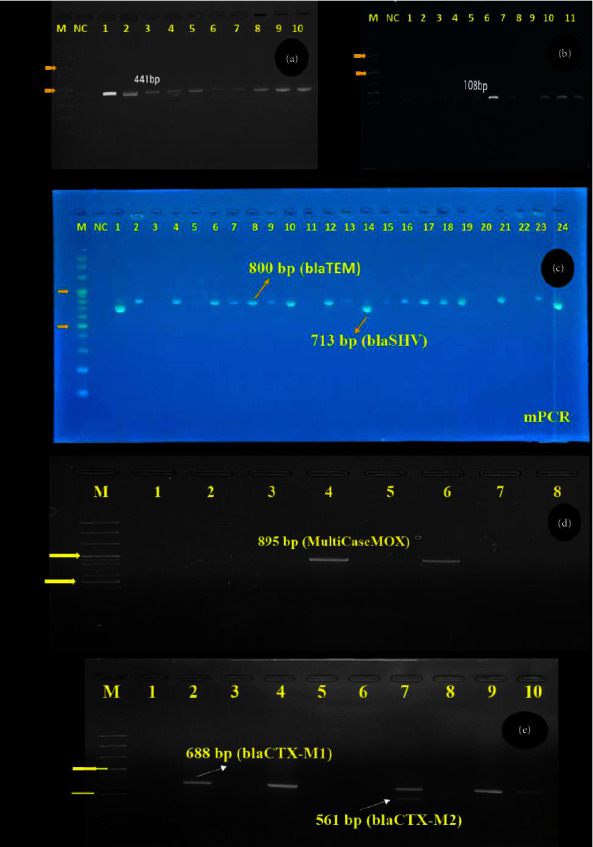
(a–e) Electrophoresis on 1.5% agarose gel showing specific amplified band of *Klebsiella* spp. and ESBL genes amplification by both Uni-PCR and multiplex PCR; lane M: 100 bp marker DNA; lane NC: control −ve; (a) lane (1–10) reaction specific (+ve) for *gyrA* gene (441 bp) of *Klebsiella* spp.; (b) *rpoB* (108 bp) for *K. pneumoniae*; (c–e) multiplex PCR for ESBL genes.

The study data, outcomes, and the interrelation among explanatory variables and the outcome variable (*K. pneumoniae* positive and negative and MDR yes/no) are comprehensively depicted in a Sankey diagram (Figure 2). According to the molecular detection through PCR, specific bands for the *gyrA* gene were detected for *Klebsiella* spp. and for the *rpoB* gene for *K. pneumoniae* (Figures 3(a) and 3(b)).

### 3.1. Prevalence and Geographic Distribution of *Klebsiella* spp

Out of the 225 samples, 65 isolates (28.89%) tested positive for *Klebsiella* spp. through molecular detection via PCR (Table 2 and Figure 3(a)), with *K. pneumoniae* accounting for 75.4% (49/65) of these positive isolates (Figure 3(b)). The prevalence of *Klebsiella* spp. varied significantly (*p* < 0.05) among the different upazilas of Sylhet, Bangladesh. The highest prevalence was observed in Fenchuganj (58.3%; 7/12), followed by Companiganj (50%; 7/14) and Kanaighat (50%; 6/12). In contrast, the lowest prevalence was recorded in Bishwanath (11.1%; 2/18) and Osmani Nagar (11.76%; 2/17). The detailed distribution of *Klebsiella* spp. is presented in Table 2.

### 3.2. Antibiogram Profiling of *K. pneumoniae*

**Figure 4:**
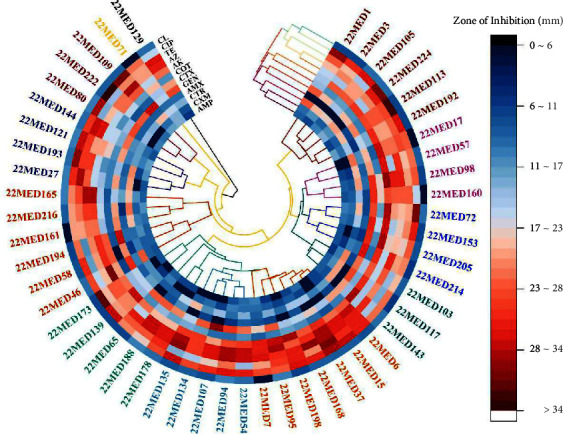
Heat map with dendrogram showing the sensitivity pattern of antibiotics with relations among the sample tested.

Out of the 49 *K. pneumoniae*-positive isolates, all exhibited complete resistance (100%) to ampicillin, amoxicillin, cefuroxime, cefotaxime, and colistin, as per the CLSI standards (Table 3 and Figure 4). In contrast, the highest sensitivity was observed with gentamicin (97.95%), followed by ciprofloxacin (85.71%), tetracycline (83.67%), and trimethoprim-sulfamethoxazole (81.63%).

All 49 isolates (100%) of *K. pneumoniae* demonstrated multidrug resistance (MDR, defined as resistance to ≥3 classes of antibiotics), each surpassing a multiple antibiotic resistance (MAR) index value of 0.20 (Table 4). The most frequent resistance pattern, observed in the highest number of isolates (*n* = 43), involved resistance to six antibiotics: ampicillin, amoxicillin, cefuroxime, cefotaxime, ceftriaxone, and colistin (AMP-AMX-CXM-CTX-CTR-CL), as shown in Table 4.

### 3.3. ESBL Phenotype-Genotype Pattern

**Figure 5:**
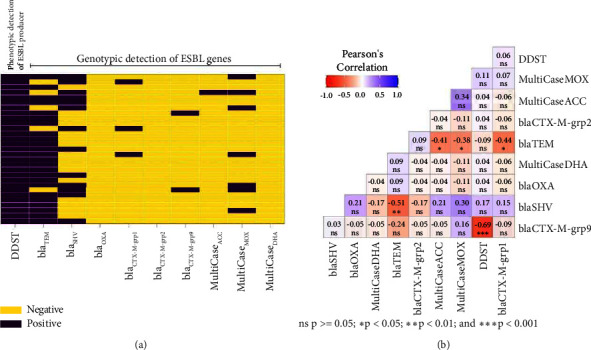
(a) Distribution of ESBL encoding genes (genotypically) and ESBL producer phenotypically among the positive isolates of *K. pneumoniae*. (b) Pearson correlation coefficients showing the associations between phenotype and genotype of ESBL genes. The level of significance is 0.05 (2-tailed). ^∗^*p* < 0.05; ^∗∗^*p* < 0.01; ^∗∗∗^*p* < 0.001; DDST: double-disk synergy test.

Only the *K. pneumoniae*-positive isolates were screened for both phenotypic and genotypic detection of ESBL genes. According to the results of DDST, 29 out of 49 *K. pneumoniae*-positive samples (59.2%) were phenotypically identified as ESBL producers (Figure 5(a)). Genotypically, the study revealed the presence of *bla*_TEM_,  *bla*_SHV_,  *bla*_CTX‐M‐grp1_,  *bla*_CTX‐M‐grp9_, *MultiCase*_ACC_, and *MultiCase*_MOX_ in *K. pneumoniae*-positive isolates (Figures 3(c), 3(d), and 3(e) and Table 5). Specifically, *bla*_TEM_ was the most frequently identified gene, occurring in 82.8% (24/29) of ESBL producer isolates, followed by *bla*_SHV_ at 44.8% (13/29). Notably, *bla*_CTX‐M‐grp1_ and *bla*_CTX‐M‐grp9_ genes were found in 10.3% (3/29) and 6.9% (2/29) of the isolates, respectively. In addition, one isolate (3.4%) was positive for *MultiCase*_ACC_, while 7 isolates (24.1%) were positive for *MultiCase*_MOX_.

Almost all ESBL producers harbored the *bla*_TEM_ gene, with most genes detected phenotypically in positive producers indicating a positive correlation between ESBL producers and ESBL genes. However, some genes did not exhibit phenotypic expression in the DDST results (Figures 5(a) and 5(b)). A significantly negative correlation was found between *bla*_TEM_ and *bla*_CTX‐M‐grp1_ (*r*_*s*_ = −0.44; *p* < 0.05) and *bla*_CTX‐M‐grp2_ (*r*_*s*_ = 0.04; *p* < 0.05).

## 4. Discussion

*Klebsiella pneumoniae* is an opportunistic pathogen that can colonize both humans and animals and is frequently found as a contaminant in retail meat [30]. The discovery of antibiotic-resistant *Klebsiella* spp. in food may enable public health professionals to develop methods that are more effective in minimizing contamination.

Our study revealed the significant presence of multidrug-resistant (MDR) *K. pneumoniae* and the prevalence of various ESBL genes in retail cattle meat in Sylhet, Bangladesh. A substantial portion of *K. pneumoniae* isolates (100%) demonstrated complete resistance to key antibiotics and the detection of *bla*_CTX‐M‐grp1_ (14.29%) and *bla*_CTX‐M‐grp9_ (4.08%) genes highlights the genetic diversity contributing to ESBL production. The findings of this study are crucial as they reflect the widespread occurrence of MDR and ESBL-producing *K. pneumoniae* in the retail meat supply, posing a significant public health risk. The presence of *bla*_CTX‐M_ genes suggests a potential for horizontal gene transfer and increased resistance, necessitating stringent surveillance and control measures to mitigate the spread of resistant strains in the food chain.

The overall occurrence of *Klebsiella* spp. in retail beef was 28.89% in the Sylhet district, a figure closely resembling the results of a study conducted in Greece (35.6%) [31] and slightly surpassing those of a study in Egypt (17.3%) [32]. This discrepancy could be attributed to the diverse hygienic and sanitary practices maintained during the slaughtering and selling processes in different countries. However, the present study revealed that the frequency of positive isolates was 75.4% (49/65) for *K. pneumoniae*, which is higher than the results of another study in 2020 [33]. Their investigation focused on testing raw and ready-to-eat retail food throughout Singapore and also reported a 27% prevalence of *K. pneumoniae* when examining samples from chicken and pork liver. This similarity in findings highlights a consistent presence of *K. pneumoniae* across different food sources.

Furthermore, the prevalence rate in our study is slightly lower than that reported in studies conducted in Greece (81.8%) and the North-west province of South Africa (32%) [31, 34]. Conversely, our findings are considerably higher when compared to research conducted in eastern China (13.8%) involving the analysis of fresh raw chicken meat [35].

The observed variation in the prevalence of *K. pneumoniae* across different studies and regions can be attributed to several factors. First, the differences in sampling methods and sample types play a crucial role; for instance, our study focused on retail cattle meat, whereas other studies examined chicken, pork liver, or mixed food sources. The microbiological quality and handling of these different food types can significantly influence the presence of pathogens. In addition, regional disparities in farming practices, antibiotic usage, and hygiene standards contribute to the variation in *K. pneumoniae* prevalence. In regions with stringent biosecurity measures and antibiotic regulations, lower prevalence rates might be observed. Conversely, in areas where antibiotic misuse is common, such as in parts of Greece and the North-west province of South Africa, higher prevalence rates may be expected [31, 34]. Environmental factors, including climate and local flora and fauna, also impact bacterial distribution and survival. The socioeconomic status and public health infrastructure of a region further influence food safety standards, affecting the presence and detection of *K. pneumoniae* [36]. Finally, genetic differences among *K. pneumoniae* strains in different geographical locations can result in varied adaptability and resistance profiles, contributing to the observed inconsistencies. These multifactorial influences underscore the complexity of microbial epidemiology and the need for region-specific interventions to manage foodborne pathogens effectively.

Our investigation revealed extensive antibiotic use in the cattle farming system of Bangladesh, driven by the elevated resistance levels (100%) of *K. pneumoniae* to crucial antibiotics, including amoxicillin, ampicillin, cefuroxime, and colistin. The findings are closely aligned with a study [37] where ampicillin and cefuroxime show maximum resistance. In our study, *K. pneumoniae* demonstrates high susceptibility to gentamicin (97.95%), tetracycline (83.67%), ciprofloxacin (85.71%), and trimethoprim-sulfamethoxazole (81.63%). However, in another study, it is depicted that gentamicin and ciprofloxacin are resistant to *K. pneumoniae* [38], whereas gentamicin, ciprofloxacin, and trimethoprim-sulfamethoxazole are moderately sensitive to *Klebsiella* portrayed by another study [39]. Our findings highlight a clear and alarming trend toward increasing resistance to previously susceptible antibiotics due to the indiscriminate use and selective pressure of antimicrobials worldwide.

Furthermore, the global dissemination of ESBL-producing bacteria poses a significant threat to both human and animal ecosystems. This investigation identified the presence of ESBL resistance genes, specifically *bla*_TEM_ and *bla*_SHV_, in *Klebsiella* spp. while *bla*_OXA_ was not detected. In the present study, the prevalence of the *bla*_SHV_ gene was 44.8% in cattle meat, and the *bla*_TEM_ gene was found at a rate of 82.8%. Interestingly, these results diverge somewhat from another study, which reported *bla*_SHV_ genes at a rate of 71.4% and *bla*_TEM_ genes at a rate of 72.2% [40].

In the North-west province of South Africa, the frequency of *bla*_TEM_ (22.9%) was notably higher, and *bla*_SHV_ (44.8%) exhibited a nearly equivalent percentage [34]. Conversely, another study found a higher prevalence of *bla*_TEM_ (94%) followed by *bla*_SHV_ (39%) [41]. Meanwhile, in Korea, a study highlighted companion animals with the highest percentage of *bla*_TEM_ (100%) and *bla*_SHV_ (94.1%) [42]. Interestingly, the other class of ESBL genes, *bla*_CTX‐M‐grp1_ (10.3%), *bla*_CTX‐M‐grp9_ (6.9%), *MultiCase*_ACC_ (3.4%), and *MultiCase*_MOX_ (24.1%), was also detected in our study. A previous study showed *bla*_CTX‐M‐grp1_ (36.5%) was detected in Trinidad and Tobago [43], which is higher than the current study.

The global dissemination of ESBL-producing bacteria poses a significant threat to both human and animal ecosystems [44, 45]. In this study, 59.2% (29/49) of the isolates were identified as ESBL producers phenotypically, while nearly all isolates were positive for ESBL genes genotypically, despite having no specific correlation between the phenotypic and genotypic results. Some isolates were phenotypically negative for ESBL production but were genotypically positive for ESBL genes. This discrepancy highlights the complexity of ESBL detection and the potential limitations of relying solely on phenotypic methods. The presence of ESBL genes does not necessarily translate to their expression. Various factors, including regulatory mechanisms and environmental conditions, can influence gene expression [9]. In addition, mutations in the regulatory regions of these genes can impact their expression [10]. Molecular methods such as PCR can detect low levels of ESBL genes, whereas phenotypic methods such as DDST may not detect low-level genetic expression, leading to differing findings and phenotypic expression of ESBL genes [46].

The variations in regional distribution of ESBL genes, leading to differing results across studies, may be linked to the extensive use of ESBL antibiotics or variations in the detection methods employed. Our findings underscore that cattle meat serves as a potential source of contamination with antimicrobial-resistant *K. pneumoniae* posing a potential public health risk. The results emphasize the need for heightened awareness among meat handlers regarding their role in either spreading or controlling the disease-causing bacteria. The significance of adopting sound hygiene and handling practices during meat processing is emphasized by this study as a means to reduce the risk of potential pathogen acquisition.

## 5. Conclusion

This study underscores the significant presence of multidrug-resistant (MDR) and extended-spectrum *β*-lactamase (ESBL)-producing *K. pneumoniae* in retail cattle meat from Sylhet, Bangladesh. With 100% of the isolates exhibiting complete resistance to critical antibiotics such as ampicillin, amoxicillin, cefuroxime, cefotaxime, and colistin, and a substantial prevalence of ESBL genes including *bla*_TEM_ and *bla*_SHV_, the findings highlight an alarming trend towards increasing antibiotic resistance. The discrepancy between phenotypic and genotypic detection of ESBL producers suggests limitations in phenotypic methods and emphasizes the necessity of molecular diagnostics for accurate detection. The study also reveals significant geographic variations in the prevalence of *K. pneumoniae*, influenced by regional hygienic practices, antibiotic usage, and detection methods. These findings call for stringent surveillance and control measures, improved hygiene practices, and responsible antibiotic use to mitigate the spread of resistant strains in the food chain. Enhanced awareness and education among meat handlers are crucial to prevent contamination and ensure food safety.

## Figures and Tables

**Table 1:** Primer designing for identification of ESBL-resistant *K*. *pneumoniae* isolated from cattle meat.

Primers	Targeted genes/organism	Primer sequences	Amplicon size (bp)	Reference
*gyrA*	*Klebsiella* spp.	F-CGCGTACTATACGCCATGAACGTA	441	[23]
		R-ACCGTTGATCACTTCGGTCAGG		

*rpoB*	*K*. *pneumoniae*	F-CAACGGTGTGGTTACTGACG	108	[24]
		R-TCTACGAAGTGGCCGTTTT		

*bla*_TEM_	*TEM-1* and *2*	F-CATTTCCGTGTCGCCCTTATTC	800	[25]
		R-CGTTCATCCATAGTTGCCTGAC		

*bla*_SHV_	*SHV-1*	F-AGCCGCTTGAGCAAATTAAAC	713	
		R-ATCCCGCAGATAAATCACCAC		

*bla*_OXA_	*OXA-1,4* and *30*	F-GGCACCAGATTCAACTTTCAAG	564	
		R-GACCCCAAGTTTCCTGTAAGTG		

*bla*_CTX‐M‐grp1_	*CTX-M-1, CTX-M-3,* and *CTX-M-15*	F-TTAGGAAATGTGCCGCTGTA	688	
		R-CGATATCGTTGGTGGTACCAT		

*bla*_CTX‐M‐grp2_	*CTX-M-2*	F-CGTTAACGGCACGATGAC	404	
		R-CGATATCGTTGGTGGTACCAT		

*bla*_CTX‐M‐grp9_	*CTX-M-9* and *CTX-M-14*	F-TCAAGCCTGCCGATCTGGT	561	
		R-TGATTCTCGCCGCTGAAG		

*MultiCase*_ACC_	*ACC-1* and *ACC-2*	F-CACCTCCAGCGACTTGTTAC	346	
		R-GTTAGCCAGCATCACGATCC		

*MultiCase*_MOX_	*MOX-1, MOX-2, CMY-1, CMY-8 to CMY-11,* and *CMY-19*	F-GCAACAACGACAATCCATCCT	895	
		R-GGGATAGGCGTAACTCTCCCAA		

*MultiCase*_DHA_	*DHA-1* and *DHA-2*	F-TGATGGCACAGCAGGATATTC	997	
		R-GCTTTGACTCTTTCGGTATTCG		

**Table 2:** Prevalence of *Klebsiella* spp. in cattle meat in different locations (Sylhet District) of Bangladesh.

Location	*x*	No. of samples tested	Prevalence (%)	95% CI	*p* value
Balaganj	3	14	21.43	4.66–50.80	0.03
Beanibazar	4	12	33.33	9.92–65.11	
Bishwanath	2	18	11.11	1.37–34.71	
Companiganj	7	14	50.00	23.04–76.96	
Fenchuganj	7	12	58.33	27.67–84.83	
Golapganj	4	15	26.67	7.79–55.10	
Gowainghat	6	14	42.86	17.66–71.14	
Jaintapur	2	13	15.38	1.92–45.45	
Kanaighat	6	12	50.00	21.09–78.91	
Osmani Nagar	2	17	11.76	1.46–36.44	
Dakshin Surma	6	21	28.57	11.28–52.17	
Sylhet Sadar	10	50	20.00	10.03–33.72	
Zakiganj	6	13	46.15	19.22–74.87	
Total	65	225	28.89	22.97–38.81	

n/N, Sample positive/sample tested; CI, confidence Interval.

**Table 3:** Antibiogram profiling of selected antibiotics against isolated *K. pneumoniae* (*n* = 49) from retail cattle meat by the disk diffusion method.

Antibiotics	Susceptible (S)		Intermediate (I)		Resistant (R)	
	No. of isolates	%	No. of isolates	%	No. of isolates	%
Penicillin						
Ampicillin (AMP, 10 *μ*g)	0	0.00	0	0.00	49	100.00
Amoxicillin (AMX, 10 *μ*g)	0	0.00	0	0.00	49	100.00
Aminoglycosides						
Gentamicin (GEN, 10 *μ*g)	48	97.95	1	2.04	0	0.00
Amikacin (AK, 10 *μ*g)	33	67.34	16	32.66	0	0.00
Cephalosporins						
Cefuroxime (CXM, 30 *μ*g)	0	0.00	0	0.00	49	100.00
Ceftriaxone (CTR, 30 *μ*g)	0	0.00	6	12.25	43	87.75
Cefotaxime (CTX, 30 *μ*g)	0	0.00	0	0.00	49	100.00
Tetracyclines						
Tetracycline (TE, 30 *μ*g)	41	83.67	8	16.33	0	0.00
Fluoroquinolones						
Ciprofloxacin (CIP, 5 *μ*g)	42	85.71	7	14.29	0	0.00
Polymyxins						
Colistin (CL, 30 *μ*g)	0	0.00	0	0.00	49	100.00
Sulfonamides						
Trimethoprim-sulfamethoxazole (COT, 1.25/23.75 *μ*g)	40	81.63	9	18.37	0	0.00
Monobactams						
Aztreonam (AZ 10 *μ*g)	34	69.38	15	30.62	0	0.00

**Table 4:** Phenotypic pattern of multidrug-resistant (MDR) isolates and multiple antibiotic resistance index (MARI) of meat.

Phenotypic pattern	No. of resistant antibiotics (class)	n/MDR/P	MDR (%)	MAR index
AMP-AMX-CXM-CTR-CTX-CL	6 (03)	43	100% (49/49)	0.50
AMP-AMX-CXM-CTX-CL	5 (03)	06		0.42

MDR, multidrug resistant; MAR index, multiple antibiotic resistance index; n/MDR/P, no. of multidrug-resistant phenotype; CTX, cefotaxime; CXM, cefuroxime; CTR, ceftriaxone; AMX, amoxicillin; AMP, ampicillin; CL, colistin.

**Table 5:** The frequency of ESBL genes among DDST-positive *K. pneumoniae* (*n* = 29) isolated from retail cattle meat.

Detected genes	*n* (%)	95% CI
*bla*_TEM_	24 (82.8%)	64.2–94.2
*bla*_SHV_	13 (44.8%)	26.5–64.3
*bla*_OXA_	0 (0%)	N/A
*bla*_CTX‐M‐grp1_	3 (10.3%)	2.2–27.4
*bla*_CTX‐M‐grp2_	0 (0%)	N/A
*bla*_CTX‐M‐grp9_	2 (6.9%)	0.85–22.8
*MultiCase*_ACC_	1 (3.4%)	0.09–17.8
*MultiCase*_MOX_	7 (24.1%)	10.3–43.5
*MultiCase*_DHA_	0 (0%)	N/A

N/A, not applicable; *n*, number of positive isolates; CI, confidence Interval.

## Data Availability

The data used to support the findings of this study are available from the corresponding author, DC, upon reasonable request.
